# *In vitro* antiproliferation activity of *Typhonium flagelliforme* leaves ethanol extract and its combination with canine interferons on several tumor-derived cell lines

**DOI:** 10.14202/vetworld.2020.931-939

**Published:** 2020-05-19

**Authors:** Bambang Pontjo Priosoeryanto, Riski Rostantinata, Eva Harlina, Waras Nurcholis, Rachmi Ridho, Lina Noviyanti Sutardi

**Affiliations:** 1 Division of Veterinary Pathology, Faculty of Veterinary Medicine, IPB University, Bogor, Indonesia; 2 Tropical Biopharmaca Research Center, IPB University, Bogor, Indonesia; 3 Department of Biochemistry, Faculty of Mathematics and Natural Sciences, IPB University, Bogor, Indonesia; 4 Faculty of Pharmacy, Gunadarma University, Depok, Indonesia; 5 Division of Pharmacy, Faculty of Veterinary Medicine; IPB University, Bogor, Indonesia

**Keywords:** antiproliferation, antiangiogenesis, canine interferons, ethanol extract, tumor cell lines, *Typhonium flagelliforme*

## Abstract

**Background and Aim:**

Tumor disorder is one of the degenerative diseases that affected human and animals and recently is tend to increase significantly. The treatment of tumor diseases can be performed through surgical, chemotherapy, radiotherapy, biological substances, and herbs medicine. *Typhonium flagelliforme* leaves extract known to have an antiproliferation activity, while interferons (IFNs) one of the cytokines that first used as an antiviral agent was also known to have antitumor activity. Nowadays, the treatment of tumors using a traditional way, including the use of herbal substances, becomes popular. Some limitations of the antitumor activity due to resistant development of the cell to some substances were one of the problems on why the treatment of cancer was unsuccessful. This study aimed to elaborate the synergistic effect on the antiproliferation and anti-angiogenesis activities of the combinations between *T. flagelliforme* leaves ethanol extract and canine natural (natural canine IFN [nCaIFN]) and recombinant (recombinant canine IFN [rCaIFN]) IFNs on tumor-derived cell lines to find the new potential antitumor substances.

**Materials and Methods:**

The extraction of *T. flagelliforme* leaves was performed using the maceration method and followed by phytochemical screening assays. According to the result of LC_50_ by the brine shrimp lethality test, the dose used for *T. flagelliforme* extract was 120 ppm while the dose of IFNs was 10^2^ U/ml. The tumor-derived cell lines (canine squamous cell carcinoma [CSCC], canine mammary gland benign mixed tumor/MCM-IPB-B3, and feline squamous cell carcinoma [FSCC]) and normal rabbit endothelial cells were cultured and maintained on Dulbecco’s Modified Eagle’s Medium DMEM/Ham-F12 medium supplemented with 10% fetal calf serum, antibiotic, and antifungal. The antiproliferation activity was assayed by calculated the total cell number after treated with the tested substances. The antiangiogenesis assay was performed using *in vitro* method on rabbit normal endothelial cells and *in ovo* using chicken chorioallantoic membrane (CAM).

**Results:**

The phytochemical screening test of the *T. flagelliforme* leaves ethanol extract indicated that the compound consisted of flavonoid, steroid, and tannin. The antiproliferation activity was increased in the combination of substances compared to the single exposure of each substance on all tested tumor-derived cell lines. There was no significantly different on the antiproliferation activity between a combination of *T. flagelliforme* with nCaIFN or rCaIFN in every single tested cell lines, but the comparison of this activity among the three tumor-derived cell lines seem that the antiproliferation activity is more effective on CSCC cell lines compared to the canine mammary gland benign mixed tumor and FSCC cell lines. A similar pattern of synergistic effect was also detected on the anti-angiogenesis activity *in vitro* using rabbit endothelial cells as well as *in ovo* assays. The most effective of the *in vitro* and *in ovo* anti-angiogenesis activity was observed on the combination substances between *T. flagelliforme* extract and rCaIFN compared to other treatments.

**Conclusion:**

There was a synergistic effect on the antiproliferation and antiangiogenesis activities of the combination between *T. flagelliforme* and canine IFNs (natural and recombinant) and this result could be developed as another alternative on the cancer treatments.

## Introduction

Tumor is a disturbance of growth characterized by excessive, abnormal, and uncontrolled proliferation of transformed or altered cells at one or more primary points within the host, and frequently at one or more metastatic sites [1]. The causes of tumor are complex and involving many factors such as carcinogenic agents, viruses, irradiation, and inflammation [2]. Tumor disorders could be affected not only humans but also animals, especially small animals such as dogs and cats. In recent year, the case of tumor disorders both in human and animal is increasing significantly [3,4]. Tumor cases in dog, which were detected from our necropsy room of the Division of Veterinary Pathology Faculty of Veterinary Medicine IPB University were counted about 22% in 1996-1998 [4]. The reported data of canine and feline tumors from Venice and Vicenza Province during 2005-2013 showed that the cases of mammary tumor were about 2744 cases [5]. The cases in Switzerland from 1955 to 2008 showed that about 63,214 dogs from 121,963 heads were diagnosed with tumors disorders [6]. Tumor cases of Golden Retriever in Dutch also showed about 4653 cases from 29,304 Golden Retriever populations during 1998-2004 [7].

The most common treatments applied for tumor cases are chemotherapy and surgical procedures. Other treatments that can be performed for tumor are the usage of biological substances and herbs medicine. Nowadays, peoples prefer to use herbs medicine for some medicinal treatments. Indonesia is a mega biodiversity country of flora and fauna. The usage of herbs plant for therapy had been done from many centuries ago, but it was not well documented [8]. One of the herbs from Indonesia that has an antitumor activity is *Typhonium flagelliforme*. This plant belongs to the family of Araceae and known as “Keladi Tikus” in Indonesian. Our previous study reported that *T. flagelliforme* leaves ethanol extract had the antiproliferation activity on tumor epithelial cells lines derived from canine acanthomatous epulis (MCA-B1) and canine mammary gland benign mixed tumor cell lines/MCM-B2 [9].

Another medicinal treatment for tumor is the usage of biological substances such as interferon (IFN). IFN is one of the cytokines, which is produced due to the response to stimulation of intracellular antigens and widely known as antiviral agents [10]. Our previous study showed that there was an antiproliferation activity of canine IFNs (CaIFN) on several tumor-derived cell lines [11]. Antiproliferation and antiangiogenesis activities of a single exposure of IFN [12,13] as well as plants extract in tumor cells sometime varied or even lack effective among the tumor cells [1]. So far, there was no study reported on the observation of the antiproliferation and antiangiogenesis activities of the combination between herbs extract and IFNs on tumor cells.

This study aimed to elaborate on the antiproliferation and antiangiogenesis activities of the combination between *T. flagelliforme* leaves ethanol extract with natural and recombinant CaIFNs (nCaIFN and rCaIFN) on tumor-derived cell lines to increase the potency of both substances on these activities.

## Materials and Methods

### Ethical approval

This study was approved by the Animal Ethics Committee, Faculty of Veterinary Medicine, IPB University, certificate No. 108A/KEH/SKE/X/2018.

### Study period and study location

The study was carried out in the Division of Veterinary Pathology, Faculty of Veterinary Medicine, IPB University and Tropical Biopharmaca Research Center, IPB University from July 2018 – April 2019.

### Extraction of *T. flagelliforme* leaves

*T. flagelliforme* plant was identified by the Research Center for Biology of Indonesian Institute of Sciences Bogor. The extraction of the plant was done according to the maceration method of our previous study [14]. Briefly, the dried leaves simplicia of *T. flagelliforme* leaves was ground to 60 mesh size. About 500 g of ground simplicia was soaked into 70% ethanol as the solvent for 24 h. The macerated simplicia was then filtered and the obtained dregs were soaked into the same solvent and then filtered. The filtrates were evaporated by rotary evaporator to get the viscous extract.

### Phytochemical screening

Phytochemical screening was performed to analyze the extract for the presence of secondary metabolite compounds, such as flavonoid, alkaloid, tannin, steroid, triterpenoid, and saponin, according to standard methods [15].

#### Alkaloid

About 40 mg of the extract was added by 2 ml of chloroform and ammonia then filtered. The filtrate was added by H_2_SO_4_ about 3-5 drops then homogenized. The obtained acid fraction was reacted with Dragendorff, Meyer, and Wagner reagents. The appearance of yellow to red (Dragendorff), white (Meyer), and brown to black (Wagner) precipitate indicated the presence of alkaloid.

#### Flavonoid

Hundred milliliters of hot water were added into 40 mg of the extract and boiled for 5 min then filtered. About 0.05 mg of Mg and 1 ml of HCl were added into 5 ml of filtrate and homogenized. The discoloration to red, yellow, or orange indicates the presence of flavonoid.

#### Tannin

Forty milligrams of the extract were added with 10 drops of 1% FeCl_3_. The presence indication of tannin was the discoloration of the solution to greenish-black color.

#### Steroid/Triterpenoid

Ten drops of CH_3_COOH and 2 drops of H_2_SO_4_ were added into 40 mg of the extract, shaken and then stand for several minutes. The presence of steroid release blue or green color, while the presence of triterpenoid release red or purple color.

#### Saponin

Forty milligrams of the extract were added with 10 ml of water then shaken for 1 min. The presence of saponin was showed by stable foam for 5 min.

### CaIFN

CaIFNs used in this study were natural (nCaIFN) and recombinant (rCaIFN) which prepared and provided by our previous study [16]. The IFNs were stocked and stored in the freezer and prepared normally by warm thawed when it will be used. The dose of IFN was 10^2^ U/ml according to our previous study [11].

### Cell culture

Cell lines used were canine squamous cell carcinoma (CSCC) and feline squamous cell carcinoma (FSCC) [16], canine mammary gland benign mixed tumor/MCM-IPB-B3 [17]. The cell lines have been diluted to be a suspension with a density of 10^6^ cells/ml and stored in liquid nitrogen until use. The frozen cell lines suspension was thawed at room temperature. The cell lines suspension was homogenized and prepared to be ready to use. The medium for cultivation of the cell line was Dulbecco’s modified Eagle’s medium (DMEM)/Ham-F12 supplemented with fetal calf serum (FCS), antibiotic, and antifungal.

### Antiproliferation assay

Cells lines were treated with three treatments, i.e., *T. flagelliforme* ethanol extract, combinations between *T. flagelliforme* ethanol extract and IFNs (nCaIFN and rCaIFN), and doxorubicin as the positive control. All treatments were in three replicates. The cell lines were cultivated onto 24-wells microplates containing complete culture medium (DMEM, FCS, antibiotic, and antifungal). The concentration of *T. flagelliforme* ethanol extract was 120 ppm which based on LC_50_ calculation from our previous study [9] and 10^2^ U/ml for CaIFNs [11]. Cultivated cells were then incubated in a 37°C incubator with 5% CO_2_ for 4 days. After the cells on the control holes were confluent, all cells in the treatment holes were then harvested and counted under a phase-contrast microscope using a hemocytometer with Trypan Blue dye exclusion. The percentage of antiproliferation activity was obtained by calculated the total number of cells using the formulation below:


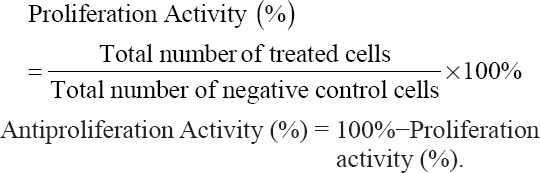


### Antiangiogenesis

#### In vitro assay

*In vitro* assay of antiangiogenesis was performed using a rabbit endothelial cell lines developed in our previous study [18]. The *in vitro* assay method of antiangiogenesis had the same method with antiproliferation. The endothelial cell lines were treated with *T. flagelliforme* ethanol extract, nCaIFN, rCaIFN, and combination between *T. flagelliforme* ethanol extract with CaIFN (nCaIFN and rCaIFN) on three replicates for each treatment.

#### Chorioallantoic membrane (CAM) assay

Antiangiogenesis assay was applied using a chicken CAM assay on 5 days old of embryonic eggs. The embryonic eggs were perforated on the air sac region for injected the treatment substances. The hole was closed with a wax to make the embryonic development process continue. The incubation of the eggs was continue in an incubator until the observation was finished. The growth of blood vessels (angiogenesis) was observed directly by (1) candling the eggs and (2) breaking the eggshell. Both observations were performed every 3 days until day 14^th^ [19]. All embryonic eggs were euthanized according to the procedure of AVMA Guidelines [20].

## Results

### Extraction of *T. flagelliforme* leaves

**Figure-1:**
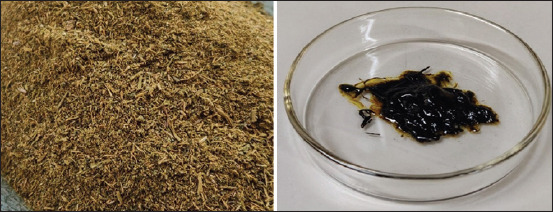
*Typhonium flagelliforme* leaves simplicia (left) and ethanol extract (right).

After maceration and evaporation of the *T. flagelliforme* simplicia, the whole extract on the form of a brown-black sticky substance (Figure-1) was collected on sterile plates and stored in the refrigerator.

### Phytochemical screening

The phytochemical assay of the *T. flagelliforme* leaves showed that this ethanol extract was contained flavonoid, tannin, and steroid, the detailed result is shown in Table-1.

**Table-1:** The phytochemical screening of *T. flagelliforme* leaves ethanol extract.

Phytochemical compounds	*T. flagelliforme* leaves ethanol extract
Alkaloid	
Dragendorff	−
Meyer	−
Wagner	−
Flavonoid	+
Saponin	−
Steroid	+
Tannin	+
Triterpenoid	−

T. flagelliforme=Typhonium flagelliforme

### Antiproliferation assay

**Figure-2:**
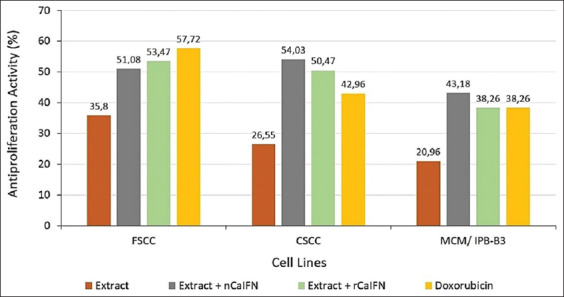
Antiproliferation activity of combination between *Typhonium flagelliforme* leaves ethanol extract with natural canine interferon and recombinant canine interferon on feline squamous cell carcinoma, canine squamous cell carcinoma, and MCM/IPB-B3 tumor-derived cell lines.

The antiproliferation activity of the tested substances showed that there was an effect on the growth inhibition of all tumor cell lines (Figure-2). In the FSCC cells, the highest antiproliferation activity with a percentage of 53.47% was achieved by the combination between *T. flagelliforme* ethanol extract and rCaIFN. In the CSCC cell line, the highest antiproliferation activity was achieved in the combination between *T. flagelliforme* ethanol extract and nCaIFN with a percentage of 54.03%, this result even higher than doxorubicin treated cells. In the MCM/IPB-B3 cells, the highest activity was achieved in the combination between *T. flagelliforme* ethanol extract and nCaIFN with a percentage of 43.18%, this activity was slightly high compared to the doxorubicin-treated cells.

Statistical analysis (Table-2) of the antiproliferation activity between single extract and combination treatments exposed to all cell lines shows a significant difference (p<0.05), while the combination treatment was not different between the cell lines (p>0.05). On the other hand, all treatments were significantly different between the cell lines (p<0.05).

**Table-2:** Antiproliferation activity of the treatments in each cell lines.

Treatment	Average antiproliferation activity on cell lines (%)		

	FSCC	CSCC	MCM/IPB-B3
Doxorubicin	57.77^a,p^	43.10^a,q^	38.15^a,r^
*T. flagelliforme* ethanol extract+nCaIFN	51.85^a,p^	54.03^a,p^	43.18^a,q^
*T. flagelliforme* ethanol extract+rCaIFN	53.47^a,p^	41.66^a,q^	26.73^a,s^
*T. flagelliforme* ethanol extract	35.84^b,r^	26.48^b,s^	20.47^b,t^

Different letter (a, b) in the same column indicated significantly different (p<0.05), Different letter (p, q, r, s, t) in the same row indicated significantly different (p<0.05). *T. flagelliforme=Typhonium flagelliforme*, FSCC=Feline squamous cell carcinoma, CSCC=Canine squamous cell carcinoma, nCaIFN=Natural canine interferon, rCaIFN=Recombinant canine interferon

In general, the anti-proliferation activity of all combinations between *T. flagelliforme* ethanol extract and CaIFNs showed a synergistic effect on all tumor cell lines. Anti-proliferation activity on MCM/IPB-B3 cells was the lowest compared to the other two tested tumor cell lines.

### Antiangiogenesis

#### In vitro assay

**Figure-3:**
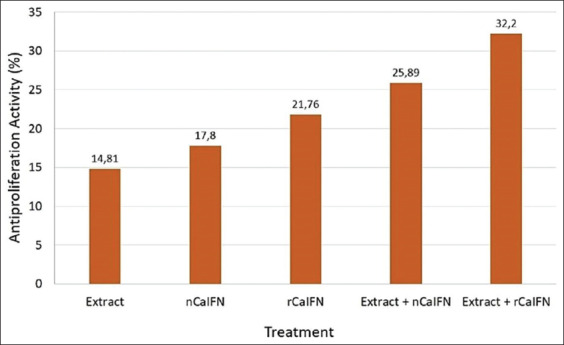
*In vitro* antiproliferation activity of *Typhonium flagelliforme* leaves ethanol extract, natural canine interferon and recombinant canine interferon single or in combinations on the rabbit endothelial cell lines.

In the *in vitro* assay using rabbit endothelial cells, there was an antiangiogenesis activity in all tested substances (Figure-3). The highest antiproliferation activity was achieved by the combination between *T. flagelliforme* ethanol extract and rCaIFN with a percentage of 32.2%. However, the lowest activity was found in the *T. flagelliforme* ethanol extract with a percentage of 14.81%. The treatment groups with single substance (*T. flagelliforme* ethanol extract or CaIFNs) showed lower activity compared to the combination treatment groups between *T. flagelliforme* ethanol extract with CaIFNs (p<0.05).

#### CAM assay

**Figure-4:**
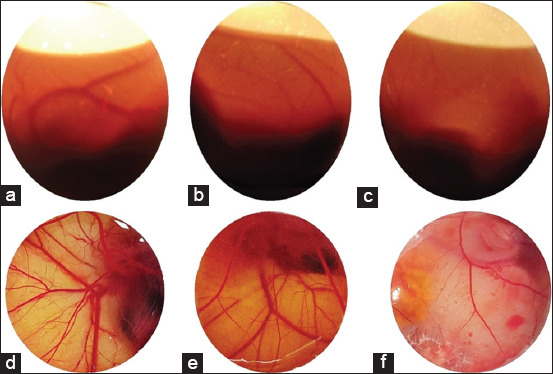
*In ovo* antiangiogenesis activity of *Typhonium flagelliforme* ethanol extract, natural canine interferon and recombinant canine interferon (rCaIFN) single or in combinations on 11 days old embryonic egg. The upper part (a-c) is the candling observation while the lower part (d-f) is the direct observation of the blood vessel development by breaking the eggshell. The left side (a and d) is the control group without treatment, the center (b and e) is treated with rCaIFN only, and the right side (c and f) is treated with the combination of *T. flagelliforme* ethanol extract and rCaIFN.

The *in ovo* antiangiogenesis assay in the chicken CAM showed a similar pattern with the *in vitro* antiangiogenesis using rabbit endothelial cells. The development of blood vessels was increased significantly in the un-treated egg compared to the treated eggs with a single substance or with the combination of substances between *T. flagelliforme* ethanol extract and CaIFNs. In the un-treated eggs, the formation of the blood vessel was very high and compact with many large blood vessels and its capillary branching. Very fine blood vessels were commonly seen on the whole of the membrane (Figure-4). In contrast to the treated eggs, especially on the group of combination between *T. flagelliforme* ethanol extract with rCaIFN, the formation of blood vessel was lower and the branching capillary was also minimum.

The moderate increase of blood vessels formation was observed both in single treatment of *T. flagelliforme* ethanol extracts and in the combination between *T. flagelliforme* ethanol extract and CaIFN (natural and recombinant IFNs).

## Discussion

IFNs are first known as a potent of antiviral agents and this was widely used in veterinary clinical practice for treated of some viral infections. IFNs also regulate other important biological properties such as cell growth and immunomodulation [21]. There were some reports of the rCaIFN production [22]. Recently, this similar CaIFN was available on the market for clinical treatment purposes. The CaIFNs use in this study was isolated in our previous study [16] and it was proven had an antiproliferation activity on tumor cell lines. Our natural CaIFN isolate was produced by the inductions of Newcastle disease virus (NDV) on normal and tumor-derived cell lines similar to those prepared by another researcher [23], while the rCaIFN was produced in the silkworm *Bombyx mori* system as other’s study [22].

There has been known that there are three types of IFNs [24]; type I IFN consisted of IFN-α, IFN-β, and IFN-Ω, these three IFNs bind to the α/β receptors 1 (IFNAR1) and IFNAR2 subunits. The type 2 IFN (IFN-γ) is known to bind to the IFN-γ receptor 1 (IFNGR1), while the type 3 IFN (IFN-λ) binds to the IFN-λ receptor 1 and also to the interleukin (IL10) receptor subunit β-heterodimeric receptor [25]. All receptors chains are binding to the binding site and will initiate the signal transduction leading to the induction of IFN genes. The binding of this cytokine to the cell surface receptors will initiate a cascade of events that induce the phosphorylation of JAK1 and TYK2 kinases, followed by the activation of the signal transducer and activation of transcription (STAT) family transcription factors [26]. The activated STAT complex induces transcription of several genes related to cell cycle arrest and apoptosis, resulting in the inhibition of cell growth and death [27].

IFNs can affect different phases of the mitotic cycle, mostly IFN therapy results in a cell cycle arrest at the G1 phase or in a prolongation and accumulation of cells in the S-phase due to the disability to complete DNA replication by the downregulation and impaired activity of cycling and cycling-dependent kinases [28]. The direct effects of IFNs were indicated by the apoptosis induction and blocking of the cell cycle. The apoptosis activity of IFNs was associated with the activation of caspases-1, -2, -3, -8, and -9. This cascade caspase activation was very important to this activity. The activation of caspase-3 was dependent on the activity of caspases-8 and -9 and the activation of caspase-8 seems to be the upstream event in IFNa-induced caspase cascade [29]. The anticancer activity of IFNs which involving its receptors was clearly shown by the study of the administration of IFN-γ which led to the inhibition of colorectal cancer cell proliferation, while the knockdown of IFNGR1 stimulated cell proliferation and colony formation potential [30]. The use of IFN-γ in the treatment of colorectal cancer has recently shown important results, against the cancer stem cell subset by inducing apoptosis both in *in vitro* and *in vivo* [31].

Our previous study on the antiproliferation activity of single exposure of this both CaIFNs (nCaIFN and rCaIFN) on three different tumor-derived cell lines indicated that they have an activity to inhibit the tumor cell proliferation, and this activity seemed more sensitive to the canine cells compared to the feline cells [11]. Besides its activity as an anticancer, some cancer cells may develop cellular tolerance that manifest to IFN-γ stimulation [32], this phenomenon could make treatment of cancer disorders by IFN will not effective. In another of our previous study, we indicated that the antiproliferation of CaIFN depended on the cell types [33]. It seems due to the presence of many receptors on the tumor cell surface resulted in a different response affinity to IFNs. This affinity of the substances was influenced by the structure, components, and mechanisms of cell metabolisms [34]. Therefore, in this study, we are looking for the possibility of the combination of IFNs with plants metabolites.

It was widely known that many plants as natural products are valuable sources of bioactive compounds and have been used in almost all cultures and communities in the world for thousands of years [35,36]. These plant extracts contain many metabolites that possess various biological activities including antitumor activity [37-39], antimicrobial, antiproliferative, and proapoptotic [40]. One of the plants that known to have an antiproliferation activity on tumor cells is *T. flagelliforme* [41]. The previous phytochemical studies indicated that several chemical constituents of the *T. flagelliforme* some of them were four pheophorbide related compounds, namely, pheophorbide-a, pheophorbide-a’, pyropheophorbide-a, and methyl pyropheophorbide-a were identified in the most active fraction on antiproliferation activity against cancer cells [42]. Several fractions of the hexane and dichloromethane extracts from *T. flagelliforme* were found to inhibit the growth of NCI-H23 non-small cell lung carcinoma cell line significantly and the D/F21 fraction was found to be the active and cancer cell line specific fraction of *T. flagelliforme* [43]. The hexane extract of *T. flagelliforme* was reported to contain saturated hydrocarbons and aliphatic acids [44], while the ethyl acetate extract was found to contain aromatic fatty acids [45].

Decreased proliferation activity of the cell lines is influenced by compounds contained in *T. flagelliforme* leaves ethanol extract which can trigger apoptosis. Apoptosis is a programmed cell death mechanism that functions to maintain the balance of cell populations in the body. The inhibition of the apoptosis is caused by proteins produced in the mitochondria, such as Bcl-2, Bcl-XL, and Mcl-1, which are classified as anti-apoptotic proteins. Flavonoids are known to be able to inhibit the proliferation activity through the intrinsic pathway of the apoptotic mechanism in several tumor cell lines. This compound can reduce the expression of anti-apoptotic proteins and increase pro-apoptotic proteins such as Bax, Bad, and Bak [46]. The flavonoid mechanism in the apoptosis process also increases the permeability of the mitochondrial membrane that can inhibit the expression of anti-apoptotic proteins and produce cytochrome C which activates the caspase so that the process of apoptosis can continue [47]. Other compounds contained in *T. flagelliforme* leaves are tannins that can inhibit the tumor cell lines proliferation by arresting the cell cycle activity in the G2/M phase and increasing apoptosis in ovarian cancer cells [48]. The arrested of the G2/M phase cell cycle also occurs by a flavonoid, and the arrested of this phase is the stage in reducing the increasing number of tumor cells that are then followed by apoptosis [49].

Angiogenesis is the process of new blood vessel formation and it was also very important in the tumor progression [50]. Formation of a new blood vessel is a necessary condition for sustained tumor growth because tumor cells take in nutrition and oxygen through the generated blood vessels, which essential for tumor cell growth [51]. Some study indicated that the growth of tumor mass will not grow more than 2-3 mm diameter without inducing their blood supply [52]. Due to a hallmark requirement of tumor growth is angiogenesis; therefore, anti-angiogenic therapy for cancer is a highly effective strategy that represents a treatment of cytostatic [53]. IFN-α was also studied for its involvement in the regulation of angiogenesis in colorectal cancer [30,54]. IFNs can inhibit angiogenesis through the downregulation of IL-8, metalloproteinase-9 (MMP-9), and basic fibroblast growth factor [54].

Plant extracts were also known to have an anti-angiogenesis activity such as *Scutellaria barbata* [55], *Jatropha curcas* [56], *Salvia triloba* [57], and others [58]. Plant metabolites such as flavones were proved to be an anti-angiogenesis agent and it was studied in some cancer cells [59]. Another study has shown that red wine polyphenolic compounds and green tea polyphenols were able to inhibit several key events of the angiogenic process such as proliferation and migration of the endothelial cells and vascular smooth muscle cells and the expression of two major proangiogenic factors, vascular endothelial growth factor and matrix MMPs [60].

*T. flagelliforme* is also has an anti-angiogenesis activity by its metabolites compound such as flavones, as shown in Table-1. Our present study indicated that when *T. flagelliforme* was exposed to the endothelial cell, it was inhibited the cell growth of rabbit endothelial cells. This feature was also in parallel when the extract was exposed to the chick embryo in CAM assay. The metabolites flavones, as well as tannin in the *T. flagelliforme* extract, could be acting as the enhancing substance. Based on the antiproliferation activity capability of *T. flagelliforme* ethanol extract, we combine these two substances to looking for an increase in the antiproliferation activity.

To the best of our knowledge, there is no study reported on the antiproliferation and anti-angiogenesis of the combination between plant extract and CaIFNs. Our present study showed that when the *T. flagelliforme* extract and CaIFNs were singly exposed to the tumor cell lines, the antiproliferation activity was lower compared to the combination forms. This phenomenon indicated that there is a synergistic effect of these two substances, even this combine mechanism is not clear yet. We still looking for the mechanism of these combine substances on how the synergistic effect was work, by the accumulation of its single effect of every substance or they work together by creating another pathway.

## Conclusion

We concluded that the combination between *T. flagelliforme* ethanol extract and CaIFNs has a synergistic effect to enhance the activity as antiproliferative and anti-angiogenesis and this combined materials have a potential to be developed as a new strategy to establish an antitumor substance.

## Authors’ Contributions

BPP and EH were involved in designing the study. RRs, WN, RRd, and LNS carried out the preparation of the extract of the herbs. RRs, BPP, and EH carried out the antiproliferation and anti-angiogenesis experiment. BPP, RRs, EH, and WN performed data collection, statistical analysis, data interpretation, and manuscript writing. BPP, EH, and RRs were involved in the monitoring of research and manuscript editing. All authors have read and approved the final manuscript.
